# 4-Nitro­benzoic acid–sulfa­thia­zole (1/1)

**DOI:** 10.1107/S1600536813034004

**Published:** 2013-12-21

**Authors:** Madhavi Oruganti, Darshak R. Trivedi

**Affiliations:** a Department of Chemistry, NITK Surathkal, Mangalore 575 025, India

## Abstract

In the crystal structure of the title compound, C_7_H_5_NO_4_·C_9_H_9_N_3_O_2_S_2_, the sulfa­thia­zole and 4-nitro­benzoic acid mol­ecules are held together by short π–π contacts between the thia­zole and nitro­benzene rings, with a centroid–centroid distance of 3.8226 (7) Å. The sulfa­thia­zole mol­ecules form dimers *via* N—H⋯N hydrogen bonds involving the thia­zole and sulfonamide moieties, owing to the fact that sulfathizole exhibits amide–imide tautomerism. The N—H (amine) groups of two sulfathiazole molecules are linked to the two S=O groups of a sulfathiazole *via* N—H⋯O hydrogen bonds. Two mol­ecules of coformer are held together by O—H⋯O hydrogen bonds. These units self-assemble, forming a three-dimensional network stabilized by (acid)C—H⋯π(sulfa­thia­zole benzene ring) inter­actions.

## Related literature   

For polymorphism in sulfa­thia­zole, see: Lagas & Lerk (1981 ▶); Blagden *et al.* (1998 ▶); Hughes *et al.* (1999 ▶); For hydrogen bonding in sulfonamides, see: Adsmond & Grant (2000 ▶). For the packing similarity of five polymorphs of sulfa­thia­zole, see: Gelbrich *et al.* (2008 ▶). For solvates of sulfa­thia­zole, see: Bingham *et al.* (2001 ▶). For co-crystals of sulfa­thia­zole, see: Shefter & Sackman (1971 ▶); Drebushchak *et al.* (2006 ▶).
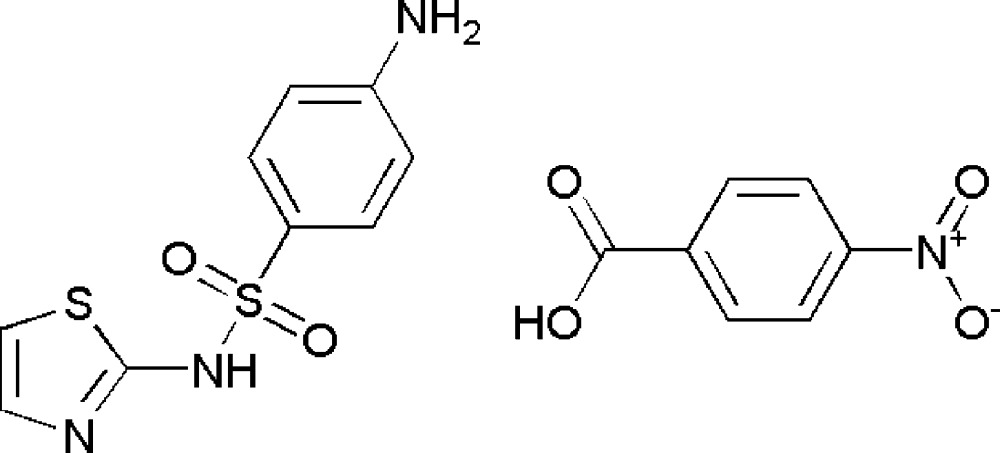


## Experimental   

### 

#### Crystal data  

C_7_H_5_NO_4_·C_9_H_9_N_3_O_2_S_2_
*M* *_r_* = 422.45
Monoclinic, 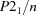

*a* = 6.6309 (2) Å
*b* = 15.0142 (6) Å
*c* = 17.7082 (7) Å
β = 94.551 (1)°
*V* = 1757.43 (11) Å^3^
*Z* = 4
Mo *K*α radiation
μ = 0.35 mm^−1^
*T* = 296 K
0.50 × 0.42 × 0.21 mm

#### Data collection  

Bruker APEXII CCD diffractometer
Absorption correction: multi-scan (*SADABS*; Sheldrick, 2004 ▶) *T*_min_ = 0.291, *T*_max_ = 0.482
17445 measured reflections
3460 independent reflections
3329 reflections with *I* > 2σ(*I*)
*R*_int_ = 0.014

#### Refinement  

*R*[*F* ^2^ > 2σ(*F* ^2^)] = 0.026
*wR*(*F* ^2^) = 0.104
*S* = 0.89
3460 reflections
309 parameters
69 restraints
All H-atom parameters refined
Δρ_max_ = 0.39 e Å^−3^
Δρ_min_ = −0.44 e Å^−3^

### 

Data collection: *SMART* (Bruker, 2001 ▶); cell refinement: *SAINT* (Bruker, 2001 ▶); data reduction: *SAINT*; program(s) used to solve structure: *SHELXS97* (Sheldrick, 2008 ▶); program(s) used to refine structure: *SHELXL97* (Sheldrick, 2008 ▶); molecular graphics: *Mercury* (Macrae *et al.*, 2008 ▶); software used to prepare material for publication: *SHELXL97* and *Mercury*.

## Supplementary Material

Crystal structure: contains datablock(s) I, bl. DOI: 10.1107/S1600536813034004/ds2237sup1.cif

Structure factors: contains datablock(s) I. DOI: 10.1107/S1600536813034004/ds2237Isup2.hkl

Supporting information file. DOI: 10.1107/S1600536813034004/ds2237Isup3.cml

Additional supporting information:  crystallographic information; 3D view; checkCIF report

## Figures and Tables

**Table 1:** Hydrogen-bond geometry (Å, °) *Cg*2 is the centroid of the C1–C6 ring.

*D*—H⋯*A*	*D*—H	H⋯*A*	*D*⋯*A*	*D*—H⋯*A*
O4—H14⋯O3^i^	1.03 (4)	1.63 (4)	2.6493 (13)	172 (3)
N1—H8⋯O1^ii^	0.82 (2)	2.22 (2)	3.0113 (15)	163.3 (18)
N1—H9⋯O2^iii^	0.838 (19)	2.326 (19)	3.0509 (15)	145.0 (17)
N3—H5⋯N2^iv^	0.897 (19)	1.96 (2)	2.8583 (15)	174.2 (16)
C14—H12⋯*Cg*2^v^	0.974 (18)	2.867 (18)	3.6648 (14)	139.8 (15)

Symmetry codes: (i) 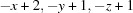; (ii) 
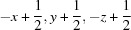
; (iii) 
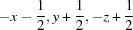
; (iv) 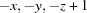; (v) 
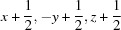
.

**Fig. 1.**
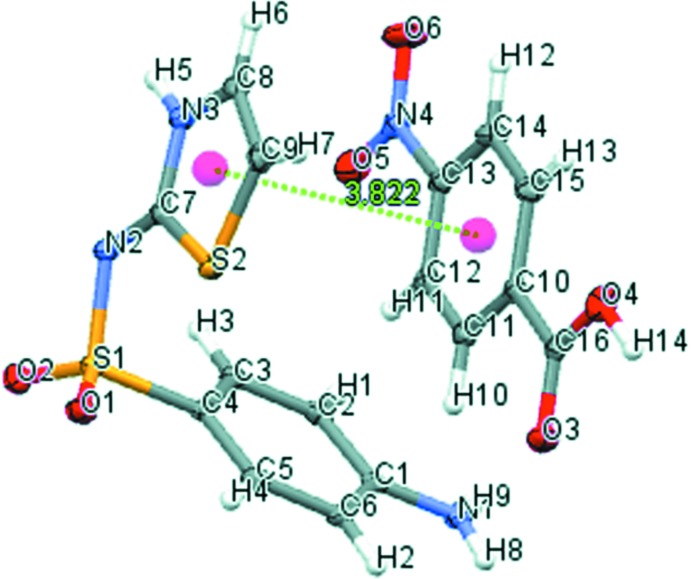
The molecular structure of the title compound showing 50% displacement ellipsoids.

**Fig. 2.**
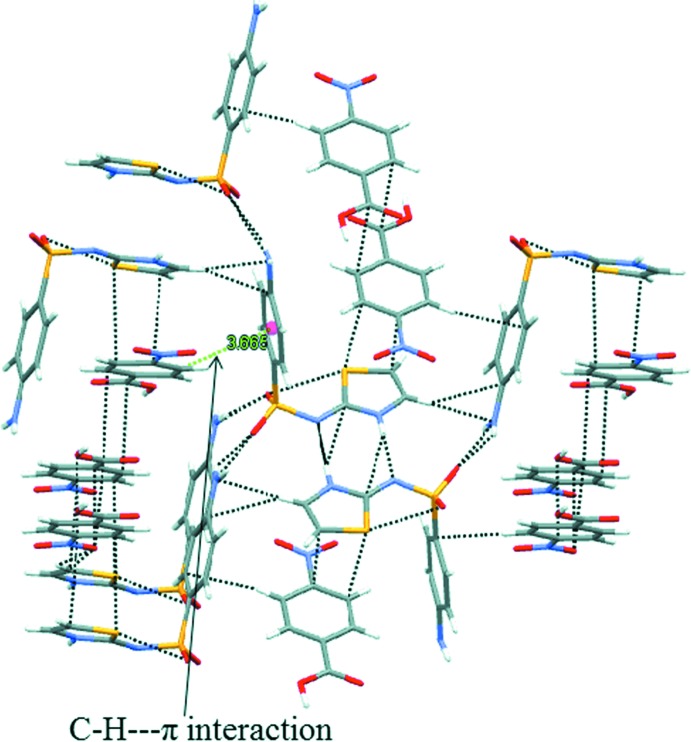
The 3D network stabilized by C—H···π interactions.

## supplementary crystallographic information

### 1. Comment

Sulfathiazole is an antimicrobial compound that belongs to the family of sulfa drugs (contains sulfonamide unit). The title compound crystallizes in *P*2_1_/*n* space group with one molecule each of sulfathiazole and 4-nitrobenzoic acid in the asymmetric unit. The two S=O (of sulfathiazole) are hydrogen bonded to the N—H (amine) of two other sulfathiazole molecules. The N of the thiazole is involved in the amide imide tautomerism thus rendering the hydrogen bond donating and accepting ability to N—H (thiazole) and N (amide). Sulfathiazole molecules thus self-assemble to form dimers *via* N—H···N hydrogen bonds [N···N 2.852 (1) Å, 〈NHN 174 (2)] involving thiazole and sulfonamide moieties. Similarly two molecules of 4-Nitrobenzoic acid interact *via* O—H···O hydrogen bonds. The whole assembly repeats to form a three-dimensional network which is stabilized by C—H (acid)···π (benzene of sulfathizole) with a distance of 3.665 Å.

### 2. Experimental

A mixture of (200 mg, 0.78 mmol) sulfathiazole and (130.9 mg, 0.78 mmol) 4-Nitrobenzoic acid were dissolved in (1:1) mixture (12 ml) of acetonitrile and ethanol, sonicated followed by mild heating. Yellow coloured needle shaped crystals were obtained in 7 days.

### Crystal data

**Table:**

C_7_H_5_NO_4_·C_9_H_9_N_3_O_2_S_2_	*Z* = 4
*M**_r_* = 422.45	*F*(000) = 872
Monoclinic, *P*2_1_/*n*	*D*_x_ = 1.600 Mg m^−^^3^
Hall symbol: -P 2yn	Mo *K*α radiation, λ = 0.71073 Å
*a* = 6.6309 (2) Å	µ = 0.35 mm^−^^1^
*b* = 15.0142 (6) Å	*T* = 296 K
*c* = 17.7082 (7) Å	Needle, yellow
β = 94.551 (1)°	0.50 × 0.42 × 0.21 mm
*V* = 1757.43 (11) Å^3^	

### Data collection

**Table:**

Bruker APEXII CCD diffractometer	3460 independent reflections
Radiation source: fine-focus sealed tube	3329 reflections with *I* > 2σ(*I*)
Graphite monochromator	*R*_int_ = 0.014
w scans	θ_max_ = 26.0°, θ_min_ = 1.8°
Absorption correction: multi-scan (*SADABS*; Sheldrick, 2004)	*h* = −8→8
*T*_min_ = 0.291, *T*_max_ = 0.482	*k* = −18→18
17445 measured reflections	*l* = −21→21

### Refinement

**Table:**

Refinement on *F*^2^	Primary atom site location: structure-invariant direct methods
Least-squares matrix: full	Secondary atom site location: difference Fourier map
*R*[*F*^2^ > 2σ(*F*^2^)] = 0.026	Hydrogen site location: inferred from neighbouring sites
*wR*(*F*^2^) = 0.104	All H-atom parameters refined
*S* = 0.89	*w* = 1/[σ^2^(*F*_o_^2^) + (0.1*P*)^2^ + 0.4859*P*] where *P* = (*F*_o_^2^ + 2*F*_c_^2^)/3
3460 reflections	(Δ/σ)_max_ = 0.001
309 parameters	Δρ_max_ = 0.39 e Å^−^^3^
69 restraints	Δρ_min_ = −0.44 e Å^−^^3^

### Special details

**Table:**

Geometry. All e.s.d.'s (except the e.s.d. in the dihedral angle between two l.s. planes) are estimated using the full covariance matrix. The cell e.s.d.'s are taken into account individually in the estimation of e.s.d.'s in distances, angles and torsion angles; correlations between e.s.d.'s in cell parameters are only used when they are defined by crystal symmetry. An approximate (isotropic) treatment of cell e.s.d.'s is used for estimating e.s.d.'s involving l.s. planes.
Refinement. Refinement of *F*^2^ against ALL reflections. The weighted *R*-factor *wR* and goodness of fit *S* are based on *F*^2^, conventional *R*-factors *R* are based on *F*, with *F* set to zero for negative *F*^2^. The threshold expression of *F*^2^ > σ(*F*^2^) is used only for calculating *R*-factors(gt) *etc*. and is not relevant to the choice of reflections for refinement. *R*-factors based on *F*^2^ are statistically about twice as large as those based on *F*, and *R*- factors based on ALL data will be even larger.

### Fractional atomic coordinates and isotropic or equivalent isotropic displacement parameters (Å^2^)

**Table:**

	*x*	*y*	*z*	*U*_iso_*/*U*_eq_	
S2	0.48584 (4)	0.124204 (19)	0.466507 (17)	0.01376 (12)	
S1	0.14759 (4)	0.067179 (19)	0.329580 (16)	0.01280 (12)	
O3	0.79216 (14)	0.45216 (6)	0.45069 (5)	0.0192 (2)	
O1	0.35756 (14)	0.06086 (6)	0.31268 (5)	0.0177 (2)	
O2	0.00325 (15)	0.01141 (6)	0.28659 (5)	0.0205 (2)	
O4	0.87828 (14)	0.45703 (6)	0.57587 (6)	0.0207 (2)	
O5	−0.09352 (14)	0.24225 (7)	0.52201 (6)	0.0238 (2)	
O6	−0.00680 (16)	0.24314 (8)	0.64238 (6)	0.0288 (3)	
N3	0.21354 (16)	0.06402 (7)	0.54483 (6)	0.0131 (2)	
N1	−0.09830 (18)	0.44403 (8)	0.28520 (6)	0.0168 (2)	
N2	0.11857 (15)	0.04399 (7)	0.41709 (6)	0.0136 (2)	
N4	0.02308 (16)	0.26183 (7)	0.57682 (6)	0.0169 (2)	
C10	0.56484 (18)	0.39184 (8)	0.53399 (7)	0.0127 (2)	
C1	−0.04137 (19)	0.35734 (8)	0.29501 (6)	0.0138 (3)	
C12	0.24664 (18)	0.32664 (8)	0.48782 (7)	0.0140 (3)	
C4	0.07641 (18)	0.17915 (8)	0.31658 (6)	0.0124 (2)	
C11	0.42711 (18)	0.36829 (8)	0.47388 (7)	0.0135 (3)	
C16	0.75894 (18)	0.43659 (8)	0.51864 (7)	0.0128 (3)	
C7	0.25113 (17)	0.07326 (7)	0.47175 (7)	0.0119 (2)	
C14	0.34332 (19)	0.33362 (9)	0.62365 (7)	0.0174 (3)	
C5	0.21340 (18)	0.24129 (8)	0.29195 (7)	0.0142 (3)	
C13	0.21044 (18)	0.30973 (8)	0.56242 (7)	0.0139 (3)	
C6	0.15541 (18)	0.32935 (8)	0.28082 (7)	0.0148 (3)	
C2	−0.17804 (18)	0.29326 (9)	0.31906 (7)	0.0154 (3)	
C3	−0.12052 (18)	0.20556 (8)	0.32984 (7)	0.0148 (3)	
C8	0.36390 (19)	0.09498 (8)	0.59690 (7)	0.0152 (3)	
C9	0.5208 (2)	0.13065 (8)	0.56467 (7)	0.0163 (3)	
C15	0.5226 (2)	0.37525 (8)	0.60900 (7)	0.0162 (3)	
H4	0.348 (2)	0.2196 (10)	0.2828 (8)	0.015 (3)*	
H2	0.241 (2)	0.3722 (10)	0.2601 (8)	0.011 (4)*	
H3	−0.214 (2)	0.1644 (11)	0.3479 (9)	0.018 (4)*	
H1	−0.309 (3)	0.3095 (11)	0.3254 (9)	0.020 (4)*	
H7	0.641 (2)	0.1555 (11)	0.5896 (9)	0.020 (4)*	
H6	0.349 (2)	0.0888 (11)	0.6484 (10)	0.017 (4)*	
H10	0.460 (3)	0.3790 (11)	0.4221 (10)	0.021 (4)*	
H13	0.611 (3)	0.3917 (11)	0.6479 (10)	0.019 (4)*	
H11	0.156 (3)	0.3092 (12)	0.4478 (10)	0.025 (4)*	
H12	0.320 (3)	0.3195 (13)	0.6759 (10)	0.029 (4)*	
H5	0.104 (3)	0.0336 (12)	0.5566 (10)	0.027 (4)*	
H9	−0.220 (3)	0.4583 (12)	0.2857 (11)	0.027 (4)*	
H8	−0.017 (3)	0.4768 (13)	0.2669 (11)	0.031 (5)*	
H14	1.001 (6)	0.493 (2)	0.561 (2)	0.109 (11)*	

### Atomic displacement parameters (Å^2^)

**Table:**

	*U*^11^	*U*^22^	*U*^33^	*U*^12^	*U*^13^	*U*^23^
S2	0.01302 (19)	0.01389 (19)	0.01468 (19)	−0.00210 (10)	0.00299 (13)	0.00102 (10)
S1	0.0164 (2)	0.01270 (19)	0.00949 (19)	−0.00064 (10)	0.00203 (13)	0.00004 (10)
O3	0.0198 (5)	0.0193 (5)	0.0197 (5)	−0.0013 (4)	0.0093 (4)	0.0007 (4)
O1	0.0197 (5)	0.0185 (5)	0.0158 (5)	0.0037 (3)	0.0074 (4)	0.0010 (3)
O2	0.0281 (5)	0.0176 (5)	0.0152 (5)	−0.0048 (4)	−0.0027 (4)	−0.0023 (3)
O4	0.0150 (5)	0.0204 (5)	0.0259 (5)	−0.0033 (4)	−0.0035 (4)	0.0017 (4)
O5	0.0178 (5)	0.0270 (5)	0.0261 (5)	−0.0070 (4)	−0.0006 (4)	0.0014 (4)
O6	0.0263 (5)	0.0393 (6)	0.0221 (5)	−0.0084 (4)	0.0090 (4)	0.0071 (4)
N3	0.0136 (5)	0.0136 (5)	0.0121 (5)	−0.0014 (4)	0.0017 (4)	0.0018 (4)
N1	0.0178 (6)	0.0163 (5)	0.0165 (6)	0.0004 (4)	0.0029 (4)	0.0025 (4)
N2	0.0153 (5)	0.0150 (5)	0.0107 (5)	−0.0025 (4)	0.0015 (4)	0.0020 (4)
N4	0.0148 (5)	0.0149 (5)	0.0216 (6)	0.0002 (4)	0.0046 (4)	0.0031 (4)
C10	0.0128 (6)	0.0112 (5)	0.0144 (6)	0.0015 (4)	0.0028 (4)	0.0004 (4)
C1	0.0182 (6)	0.0167 (6)	0.0063 (5)	−0.0011 (5)	−0.0013 (4)	−0.0005 (4)
C12	0.0141 (6)	0.0133 (6)	0.0143 (6)	0.0005 (4)	0.0000 (5)	−0.0012 (4)
C4	0.0151 (6)	0.0136 (6)	0.0084 (5)	−0.0002 (4)	0.0008 (4)	0.0007 (4)
C11	0.0153 (6)	0.0130 (6)	0.0125 (6)	0.0015 (4)	0.0028 (5)	−0.0001 (4)
C16	0.0122 (6)	0.0100 (6)	0.0163 (6)	0.0021 (4)	0.0013 (5)	−0.0005 (4)
C7	0.0135 (6)	0.0092 (5)	0.0133 (6)	0.0015 (4)	0.0020 (5)	0.0017 (4)
C14	0.0185 (6)	0.0208 (6)	0.0131 (6)	−0.0002 (5)	0.0032 (5)	0.0037 (5)
C5	0.0134 (6)	0.0187 (6)	0.0104 (5)	−0.0009 (4)	0.0006 (4)	0.0013 (4)
C13	0.0133 (6)	0.0121 (6)	0.0168 (6)	0.0014 (4)	0.0032 (4)	0.0011 (4)
C6	0.0154 (6)	0.0165 (6)	0.0125 (6)	−0.0027 (5)	0.0008 (4)	0.0034 (4)
C2	0.0138 (6)	0.0210 (6)	0.0118 (6)	0.0000 (5)	0.0030 (4)	−0.0017 (5)
C3	0.0159 (6)	0.0172 (6)	0.0118 (6)	−0.0037 (5)	0.0033 (4)	0.0003 (4)
C8	0.0183 (6)	0.0143 (6)	0.0126 (6)	0.0011 (5)	−0.0015 (5)	0.0008 (5)
C9	0.0172 (6)	0.0151 (6)	0.0164 (6)	−0.0008 (5)	−0.0012 (5)	−0.0010 (4)
C15	0.0168 (6)	0.0189 (6)	0.0127 (6)	−0.0015 (5)	−0.0010 (5)	0.0005 (4)

### Geometric parameters (Å, º)

**Table:**

S2—C9	1.7381 (14)	C1—C6	1.4122 (17)
S2—C7	1.7433 (12)	C12—C13	1.3848 (17)
S1—O2	1.4426 (10)	C12—C11	1.3896 (17)
S1—O1	1.4502 (9)	C4—C5	1.3961 (17)
S1—N2	1.6146 (10)	C4—C3	1.4022 (17)
S1—C4	1.7563 (12)	C14—C15	1.3859 (18)
O3—C16	1.2622 (16)	C14—C13	1.3890 (18)
O4—C16	1.2729 (16)	C5—C6	1.3866 (17)
O5—N4	1.2276 (16)	C2—C3	1.3798 (19)
O6—N4	1.2257 (15)	C8—C9	1.3375 (18)
N3—C7	1.3445 (16)	N1—H8	0.82 (2)
N3—C8	1.3836 (16)	N1—H9	0.84 (2)
N1—C1	1.3626 (17)	N3—H5	0.897 (19)
N2—C7	1.3294 (16)	C2—H1	0.92 (2)
N4—C13	1.4751 (16)	C3—H3	0.949 (15)
C10—C11	1.3920 (17)	C5—H4	0.976 (14)
C10—C15	1.4013 (17)	C6—H2	0.951 (14)
C10—C16	1.4958 (16)	C8—H6	0.930 (18)
C1—C2	1.4106 (17)	C9—H7	0.956 (15)
			
C9—S2—C7	91.15 (6)	N3—C7—S2	109.33 (9)
O2—S1—O1	117.43 (6)	C15—C14—C13	118.09 (11)
O2—S1—N2	104.88 (5)	C6—C5—C4	120.11 (11)
O1—S1—N2	111.93 (5)	C12—C13—C14	123.38 (11)
O2—S1—C4	109.05 (6)	C12—C13—N4	117.81 (11)
O1—S1—C4	106.74 (5)	C14—C13—N4	118.80 (11)
N2—S1—C4	106.31 (5)	C5—C6—C1	120.54 (11)
C7—N3—C8	115.34 (11)	C3—C2—C1	121.03 (11)
C7—N2—S1	120.35 (9)	C2—C3—C4	119.84 (11)
O6—N4—O5	123.71 (11)	C9—C8—N3	113.21 (11)
O6—N4—C13	118.49 (11)	C8—C9—S2	110.97 (10)
O5—N4—C13	117.80 (10)	C14—C15—C10	119.77 (12)
C11—C10—C15	120.76 (12)	C1—N1—H8	116.2 (14)
C11—C10—C16	119.79 (11)	C1—N1—H9	120.1 (13)
C15—C10—C16	119.44 (11)	H8—N1—H9	121.3 (19)
N1—C1—C2	120.80 (12)	C7—N3—H5	119.5 (11)
N1—C1—C6	120.80 (12)	C8—N3—H5	124.8 (11)
C2—C1—C6	118.40 (11)	C1—C2—H1	119.5 (10)
C13—C12—C11	117.96 (11)	C3—C2—H1	119.4 (10)
C5—C4—C3	120.06 (11)	C2—C3—H3	119.2 (9)
C5—C4—S1	120.40 (9)	C4—C3—H3	121.0 (9)
C3—C4—S1	119.53 (9)	C4—C5—H4	117.1 (9)
C12—C11—C10	120.03 (11)	C6—C5—H4	122.8 (9)
O3—C16—O4	124.74 (11)	C1—C6—H2	117.1 (9)
O3—C16—C10	118.31 (11)	C5—C6—H2	122.2 (9)
O4—C16—C10	116.95 (11)	N3—C8—H6	119.6 (9)
N2—C7—N3	120.29 (11)	C9—C8—H6	127.3 (9)
N2—C7—S2	130.38 (9)		

### Hydrogen-bond geometry (Å, º)

Cg2 is the centroid of the C1–C6 ring.

**Table:**

*D*—H···*A*	*D*—H	H···*A*	*D*···*A*	*D*—H···*A*
O4—H14···O3^i^	1.03 (4)	1.63 (4)	2.6493 (13)	172 (3)
N1—H8···O1^ii^	0.82 (2)	2.22 (2)	3.0113 (15)	163.3 (18)
N1—H9···O2^iii^	0.838 (19)	2.326 (19)	3.0509 (15)	145.0 (17)
N3—H5···N2^iv^	0.897 (19)	1.96 (2)	2.8583 (15)	174.2 (16)
C14—H12···*Cg*2^v^	0.974 (18)	2.867 (18)	3.6648 (14)	139.8 (15)

Symmetry codes: (i) −*x*+2, −*y*+1, −*z*+1; (ii) −*x*+1/2, *y*+1/2, −*z*+1/2; (iii) −*x*−1/2, *y*+1/2, −*z*+1/2; (iv) −*x*, −*y*, −*z*+1; (v) *x*+1/2, −*y*+1/2, *z*+1/2.
